# Effects of Genetically Engineered Stem Cells Expressing Cytosine Deaminase and Interferon-Beta or Carboxyl Esterase on the Growth of LNCaP Prostate Cancer Cells

**DOI:** 10.3390/ijms131012519

**Published:** 2012-09-28

**Authors:** Bo-Rim Yi, Kyung-A. Hwang, Yun-Bae Kim, Seung U. Kim, Kyung-Chul Choi

**Affiliations:** 1 Laboratory of Veterinary Biochemistry and Immunology, College of Veterinary Medicine, Chungbuk National University, Cheongju 361-763, Korea; E-Mails: rlkbsr@nate.com (B.-R.Y.); hka9400@naver.com (K.-A.H.); solar93@cbu.ac.kr (Y.-B.K.); 2 Medical Research Institute, College of Medicine, Chung-Ang University, Seoul 156-756, Korea; E-Mail: sukim2005@gmail.com

**Keywords:** human stem cells, prostate cancer, cytosine deaminase, interferon-beta, carboxyl esterase, tumor-tropism

## Abstract

The risk of prostate cancer has been increasing in men by degrees. To develop a new prostate cancer therapy, we used a stem cell-derived gene directed prodrug enzyme system using human neural stem cells (hNSCs) that have a tumor-tropic effect. These hNSCs were transduced with the therapeutic genes for bacterial cytosine deaminase (CD), alone or in combination with the one encoding human interferon-beta (IFN-β) or rabbit carboxyl esterase (CE) to generate HB1.F3.CD, HB1.F3.CD.IFN-β, and HB1.F3.CE cells, respectively. CD enzyme can convert the prodrug 5-fluorocytosine (5-FC) into the activated form 5-fluorouracil (5-FU). In addition, CE enzyme can convert the prodrug CPT-11 into a toxic agent, SN-38. In our study, the human stem cells were found to migrate toward LNCaP human prostate cancer cells rather than primary cells. This phenomenon may be due to interactions between chemoattractant ligands and receptors, such as VEGF/VEGFR2 and SCF/c-Kit, expressed as cancer and stem cells, respectively. The HB1.F3.CE, HB.F3.CD, or HB1.F3.CD.IFN-β cells significantly reduced the LNCaP cell viability in the presence of the prodrugs 5-FC or CPT-11. These results indicate that stem cells expressing therapeutic genes can be used to develop a new strategy for selectively treating human prostate cancer.

## 1. Introduction

Prostate cancer is now the most commonly diagnosed cancer in the world and is responsible for 6% of cancer deaths among men [1]. There are no distinct known causes for prostate cancer, but between 5% and 10% of cases occur in families in which individuals inherit approximately twice the risk of developing this type of cancer [2]. Prostate cancer develops when cells in the prostate gland grow in an uncontrolled manner and form a tumor mass. There are often no early symptoms of this disease, but some men experience urinary discomfort [3]. Nevertheless, aggressive tumors that grow quickly can cause severe pain and other symptoms such as difficulty in passing urine due to interrupted urine flow and frequent urination, particularly at night. This cancer can spread to bone and eventually become life threatening [4,5]. If diagnosed early, before metastasizing beyond the prostate gland, this disease can be successfully treated. However, advanced cases are very difficult to cure [6]. Surgery is the first choice of treatment for patients diagnosed with malignant prostate cancer [7]. Currently, other available therapies for prostate cancer are radical prostatectomy, irradiation therapy, palliative androgen ablation, or cryotherapy [8]. These modalities have limited efficacy for treating advanced cases, and may not be options for many patients due to a high risk of infertility and other side effects such as diarrhea, bone weakening, hot flashes or weight gain [9–11].

Genes expressing prodrug-activating enzymes (“suicidal genes”) have been recognized as an attractive tool for treating malignant prostate cancer [12,13]. Several types of these suicidal genes capable of converting non-toxic prodrugs into toxic compounds have been studied and used for gene therapy for treating various types of cancer [14,15]. Genes encoding bacterial (*Escherichia coli; E. coli*) cytosine deaminase (CD) and rabbit carboxyl esterase (CE) are most widely used to selectively and specifically kill cancer cells, thereby producing a more potent anti-tumor effect [16]. *E. coli* CD enzyme converts the prodrug 5-fluorocytosine (5-FC) into the cytotoxic agent 5-fluorouracil (5-FU), which is then converted into the more active metabolites 2′-deoxy-5-fluorouridine-5′-monophosphate (5-FdUMP) or 5-fluorouridine-5′-triphosphate (5-FUTP) [17]. Likewise, CE enzyme promotes the formation of SN-38 from irinotecan (7-ethly-10-(4-(1-piperidino)-1-piperidino)-carboyloxy-(20*S*)-camptothecin; CPT-11) by cleaving the bulky dipiperidino side chain at the carbon position [18]. Presently, CPT-11 is being tested against a variety of human malignancies and is approved for use against colon cancer [19]. The CE gene is found in many species, including humans and rabbit. When CPT-11 is administered to humans, typically less than 5% of the drug is converted into SN-38. However, rabbit CE was found to be 100–1000-fold more efficient in converting CPT-11 compared to the human isoform [14]. In gene therapy for cancer, a “double-punch system” has been devised in which interferon-beta (IFN-β) is co-expressed along with the suicide gene [20]. IFN-β belongs to type I group of IFNs that are known as potent cytokines with anti-tumor effects. Therefore, this study evaluated the use of bacterial CD and rabbit CE genes in gene-directed prodrug enzyme therapy, and assessed the effectiveness of a “double-punch system” involving the co-expression of CD and IFN-β for treating prostate cancer.

An efficient, safe, and reliable gene delivery system is critical for developing gene therapies [21]. In this respect, stem cells may be advantageous for cancer gene therapy. An extensive proliferative potential and inherent capacity to target tumors make stem cells a unique tool as cellular vehicles for targeted-gene protocols [22,23]. An efficient gene delivery system using stem cells maximizes the anti-tumor effects of transduced suicide genes or anti-tumor cytokine genes such as IFN-β [24]. Stem cell tumor tropism results from the migration of these cells to tumor cells or sites. This characteristic is due to a variety of cytokines and growth factors, including stromal cell-derived factor (SDF-1) and vascular endothelial growth factor (VEGF), produced by cancer cells or cells at a site of injury such as endothelial cells or immune cells [25]. Nevertheless, the exact mechanism underlying stem cell migration promoted by these various factors is unclear.

In the current study, we genetically engineered human neural stem cells (hNSCs) obtained from a 15-week fetal telencephalon [26]. The hNSCs were immortalized using a retrovial vector encoding v-myc and cloned. The clones were transfected with CD and/or IFN-β genes using viral vectors to produce HB1.F3.CD and HB1.F3.CD.IFN-β cells, respectively [27]. We also produced HB1.F3.CE cells expressing the rabbit CE gene to convert the prodrug CPT-11 into the toxic metabolite SN-38 [28]. Finally, we assessed the therapeutic potential of “double-punch system” of stem cell-based gene directed enzyme prodrug (CD or CE) and/or IFN-β system for treating prostate cancer in *in vitro* cellular models.

## 2. Results

### 2.1. Expression of Therapeutic Genes in the Genetically Engineered Stem Cells

**Figure 1:**
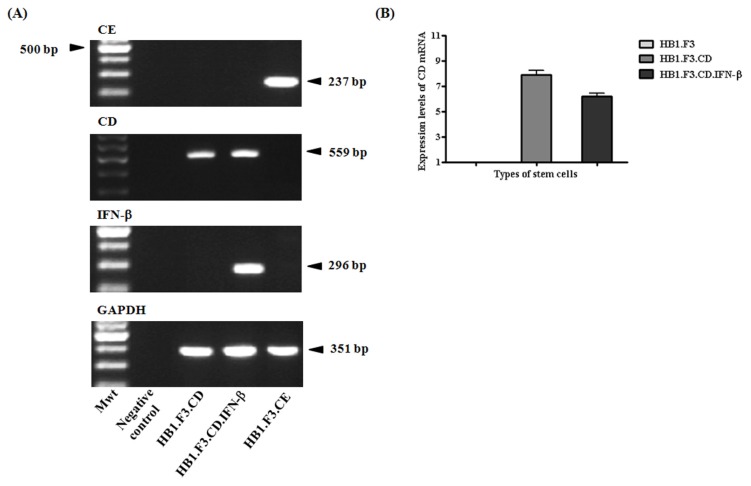
Gene expression of cytosine deaminase (CD), rabbit carboxyl esterase (CE) and interferon-beta (IFN-β) in the genetically engineered stem cells. (**A**) cDNA was produced by semi-quantitative RT-PCR to confirm the expression of CE, CD, and/or IFN-β genes; (**B**) Relative expression levels of CD gene in HB1.F3, HB1.F3.CD, and HB1.F3.CD.IFN-β cells were confirmed with quantitative real-time PCR (qRT-PCR). Mwt, molecular weight; Negative control, without cDNA template.

To confirm the expression of CE, CD, and IFN-β genes in the HB.F3.CE, HB1.F3.CD, and HB1.F3.CD.IFN-β cells, we performed a semi-quantitative reverse-transcription polymerase chain reaction (RT-PCR) assay. We confirmed the presence of the CE gene (237 bp) in HB1.F3.CE cells but not in the HB1.F3.CD or HB1.F3.CD.IFN-β cells (Figure 1A). CD gene (559 bp) expression was observed in the HB1.F3.CD and HB1.F3.CD.IFN-β cells. Only HB1.F3.CD.IFN-β cells also expressed the IFN-β gene (291 bp). The expression of glyceraldehyde 3-phosphate dehydrogenase (GAPDH, 361 bp) as a control was found in all cell lines. In addition, real-time PCR was performed to compare the expression levels of CD gene in HB1.F3.CD and HB1.F3.CD.IFN-β cells. The expression level of CD gene in HB1.F3.CD cells was similar to that of HB1.F3.CD.IFN- β cells as shown in Figure 1B. Therefore, it can be assumed that the conversion rate of 5-FC was not significantly different in HB1.F3.CD and HB1.F3.CD.IFN-β cells derived from the expressional level of CD gene.

### 2.2. Tumor-Tropic Effects of the Engineered Stem Cells against Prostate Cancer Cells

**Figure 2:**
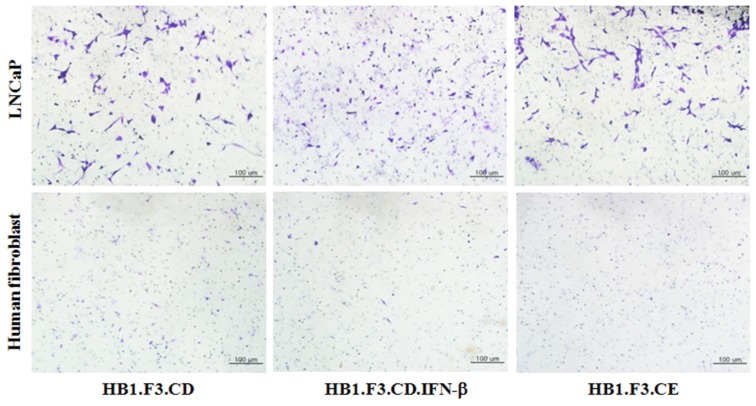
Tumor-specific migration of the genetically engineered stem cells. A transwell assay was performed in conditioned medium collected from LNCaP prostate cancer cells (treatment group) or human dermal fibroblast cells (HDF) as a control. A transwell coated with fibronectin was inserted into 24-well plates, and HB1.F3.CE, HB1.F3.CD, or HB1.F3.CD.IFN-β cells were seeded in the upper chamber of transwell. After 24-h incubation, migrated stem cells were stained with a crystal violet solution and observed with a light microscope. Magnification, 100×.

To confirm the migratory abilities of the three engineered stem cell lines, we performed a transwell-migration assay and crystal violet staining. In this experiment, the number of stem cells stained with crystal violet in an upper chamber of a transwell was higher in the conditioned media (CM)-treated well containing prostate cancer cells (LNCaP cells) than in the CM-treated well containing human fibroblasts cells after a 24-h incubation (Figure 2). In addition, there was no difference in the number of migrated stem cells regardless of transduced genes. Therefore, all types of the genetically engineered stem cells were shown to have a greater affinity for the prostate cancer cells compared to the non-tumorigenic primary cells, fibroblasts. This tumor-specific migration of the stem cells may have been due to the secretion of various cytokines and growth factors from the prostate cancer cells.

### 2.3. Chemoattractant Ligands and Receptors Regulating Stem Cells Migration

**Figure 3:**
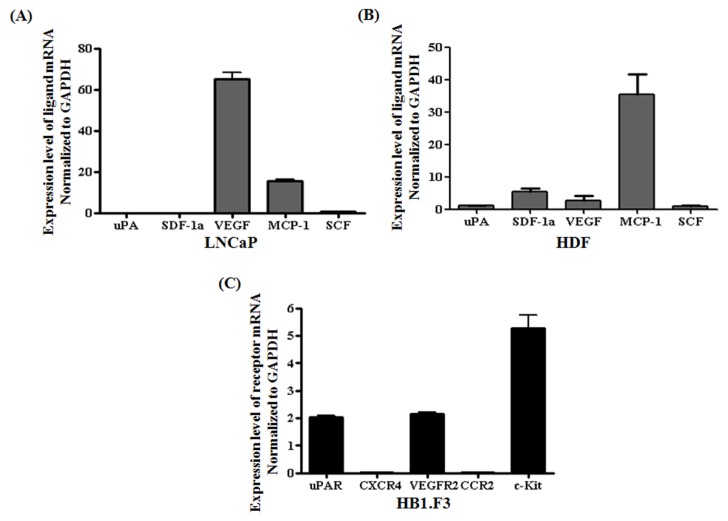
Expression of chemoattractant ligands and receptors in LNCaP prostate cancer cells, HDF and the genetically engineered stem cells (HB1.F3). Real-time PCR was performed to quantify the expression levels of related genes affecting chemoattraction. The expressions of the ligands were measured in LNCaP prostate cancer cells or HDF cells, while the expression of their receptors was assessed in the stem cells. (**A**) Expression of ligands secreted by LNCaP prostate cancer cells; (**B**) Expression of ligands secreted by HDF cells; (**C**) Expression of receptors expressed by HB1.F3.

To define the molecular mechanism by which stem cells migrate toward cancer cells, we isolated total RNA from hNSCs, HB1.F3, prostate cancer cells, and human dermal fibroblast (HDF) cells, and measured the mRNA levels of several chemoattractant ligands and receptors, including urokinase plasminogen activator and its receptor (uPA/uPAR), stem cell factor (SCF), and its receptors (c-Kit), SDF-1/CXCR4, monocyte chemotactic protein-1 (MCP-1) and its receptor (CCR2), VEGF, and VEGF-receptor 2 (VEGFR2), using quantitative real-time PCR. VEGF, MCP-1 and SCF were expressed by prostate cancer cells, but not uPA and SDF-1 (Figure 3A). HDF, as a control, weakly secreted uPA, SDF-1a, VEGF, MCP-1 and SCF for migratory effect as seen in Figure 3B. Although MCP-1 was modestly secreted among these factors in HDF cells, its receptor, CCR2, was weakly expressed in the stem cells, HB1.F3 cells (Figure 3C). On the other hand, VEGF was highly secreted in LNCaP cells and its receptor, VEGFR2, was confirmed as expressed considerably in the stem cells. Considering the expression levels of chemoattractant ligands and receptors, the interaction between VEGF and VEGFR2 may be a major cause in migratory capacity of stem cells toward LNCaP prostate cancer cells.

### 2.4. Proliferation of Prostate Cancer Cells Inhibited by CE Gene Expression in the HB1.F3.CE Cells

**Figure 4:**
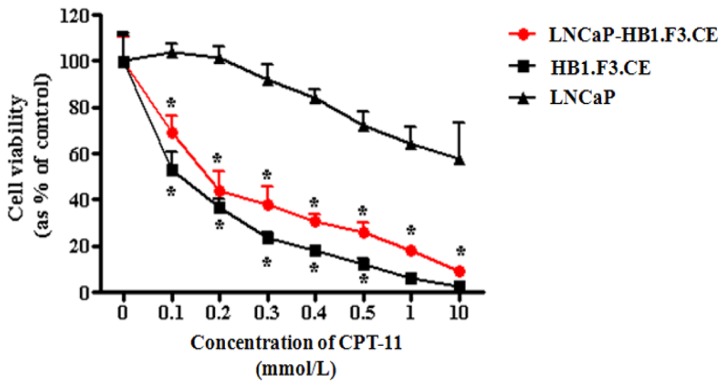
Effects of HB1.F3.CE cells and CPT-11 on prostate cancer cell viability. Prostate cancer cells were seeded in 96-well plates with or without the engineered stem cells. After 1 day of culturing, the cells were treated with various concentrations of CPT-11 (0, 0.1, 0.2, 0.3, 0.4, 0.5, 1.0, or 10.0 mmol/L) for 4 days. MTT solution was then added and the cells were incubated for 4 h at 37 °C. * *p <* 0.05 *vs*. prostate cancer cells treated with CPT-11.

We next examined how CPT-11 affects proliferation of prostate cancer cells in the absence of the HB1.F3.CE cells. CPT-11 exerts some cytotoxic effects against prostate cancer cells although it is a prodrug. We found that prostate cancer cell viability was decreased with CPT-11 at concentrations of 0.3 mmol/L to 10 mmol/L. The cytotoxic effect of CPT-11 may have been partially caused by weak expression of endogenous human CE in the prostate cancer cells. Co-culturing with HB1.F3.CE cells and treatment with CPT-11 significantly inhibited prostate cell growth by about 30% even with low concentrations of CPT-11 (0.1 mmol/L) (Figure 4). A growth of LNCaP prostate cancer cells treated with HB1.F3.CE cells was decreased in the presence of CPT-11 compared to a control in the absence of HB1.F3.CE cells. Our results demonstrated the combined antitumor effect of HB1.F3.CE cells expressing the CE gene and CPT-11, even at a low concentration (0.1 mmol/L).

### 2.5. Antitumor Effects of HB1.F3.CD or HB1.F3.CD.IFN-β Cells

**Figure 5:**
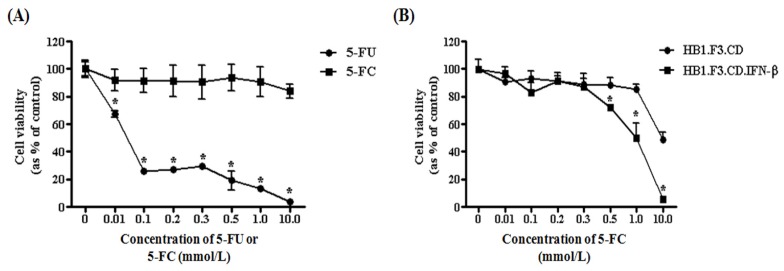
Inhibition prostate cancer cells (LNCaP cells) proliferation by HB1.F3.CD and HB1.F3.CD.IFN-β cells in the presence of 5-FC. Prostate cancer cells were seeded in a 96-well plate with or without stem cells expressing the CD and/or IFN-β gene. After incubation for 1 day, various concentrations of 5-FU or 5-FC were added to each well containing the cultured cancer and stem cells. The cells were then incubated for 4 days and cell viability was measured with an MTT assay. (**A**) The effects of treatment with various concentration of 5-FU or 5-FC (0. 0.01, 0.1, 0.2, 0.3, 0.5, 1.0, and 10.0 mmol/L) on the prostate cancer cells; (**B**) The effects of co-culturing with HB1.F3.CD or HB1.F3.CD. IFN-β cells and treatment with 5-FC on the prostate cancer cells. * *p <* 0.05 *vs*. the 5-FC treatment group or HB1.F3.CD co-cultured group.

To identify antitumor effect of CD and/or IFN-β genes, we performed cell viability assay with a co-culture of LNCaP prostate cancer cells and HB1.F3.CD or HB1.F3.CD.IFN-β cells. 5-FC, an inactive prodrug, did not have any effect on prostate cancer cell viability (Figure 5A). However, 5-FU inhibited the proliferation of prostate cancer cells by 80% when administered at a concentration of 0.1 mmol/L. To determine the therapeutic potential of the genetically engineered stem cells, HB1.F3.CD or HB1.F3.CD.IFN-β cells were co-cultured with prostate cancer cells. Cancer cell viability was decreased by the presence of both HB1.F3.CD and HB1.F3.CD.IFN-β cells with 5-FC at concentrations of 10.0 or 0.5 mmol/L, respectively (Figure 5B). HB1.F3.CD.IFN-β cells reduced the cancer cell viability to a greater degree compared to the HB1.F3.CD cells. With 0.5 mmol/L of 5-FC, HB1.F3.CD.IFN-β cells inhibited the growth of the prostate cancer cells by 25%; cancer cell viability was slightly reduced by HB1.F3.CD cells. With 10 mmol/L of 5-FC, all of the prostate cancer cells were eliminated by the HB1.F3.CD.IFN-β cells. Based on these results, we concluded that HB1.F3.CD and HB1.F3.CD.IFN-β cells exert anti-tumor effects against prostate cancer cells, and the “double punch system” involving expression of CD and IFN-β genes has strong cytotoxic effects on the cancer cells.

## 3. Experimental Section

### 3.1. Cell Lines and Cell Culture

All of stem cells, HB1.F3.CE, HB1.F3.CD, and HB1.F3.CD.IFN-β, were obtained from Chung-Ang University (Seoul, Republic of Korea) and cultured in Dulbecco’s modified Eagle’s medium (DMEM; Hyclone Laboratory, Logan, UT, USA) containing 10% fetal bovine serum (FBS; Hyclone Laboratory), 100 U/mL penicillin (Cellgrow Mediatech, Manassas, VA, USA), 100 μg/mL streptomycin (Cellgrow Mediatech), 200 mM HEPES (Invitrogen Life Technologies, Carlsbad, CA, USA), and 0.5 mL plasmocin (InvivoGen, San Diego, CA, USA). In addition, human prostate adenocarcinoma LNCaP cells were purchased from the Korean Cell Line Bank (KCLB, Seoul, Republic of Korea) and cultured in the DMEM containing 10% FBS. Primary human fibroblast cells obtained from OBM Lab (Daejeon, Republic of Korea) were maintained in the same medium. All cells were incubated at 37 °C in a humidified 5% CO_2_–95% air atmosphere.

### 3.2. Semi-Quantitative RT-PCR

Total RNA from HB1.F3.CE, HB1.F3.CD, and HB1.F3.CD.IFN-β cells was recovered with TriZol reagent (Invitrogen Life Technologies) according to the manufacturer’s protocol. Concentration of the isolated total RNA was measured with a spectrophotometer (Optizen, Mecasys, Daejeon, Republic of Korea) and 1 μg of total RNA was used to synthesize cDNA using murine leukemia virus reverse transcriptase (M-MLV RT; iNtRON Biotechnology, Sungnam, Korea), 200 pM nonamer random primer (TaKaRa Co., Ltd., Osaka, Japan), 10 mM dNTPs (iNtRON Biotechnology), 4 μL 5× RT buffer (iNtRON Biotechnology), and 10 unit RNase inhibitor (iNtRON Biotechnology) at 37 °C for 1 h. The cDNA template was amplified by PCR in a mixture of 2.5 Unit Taq polymerase (iNtRON Biotechnology), 2 μL 10× PCR buffer (iNtRON Biotechnology), 10 mM dNTPs (iNtRON Biotechnology), and 200 pmol/L reverse and forward primers specific for CE, CD, IFN-β, and GAPDH (Bioneer Corporation, Daejoen, Republic of Korea) using a PTC-100 thermocycler (MJ Research Inc., Waltham, MA, USA). PCR was performed for 30 cycles of denaturation at 95 °C for 30 s, annealing at 58 °C for 30 s, and extension at 72 °C for 30 s. The primer sequences and predicted product sizes are shown in Table 1. The PCR products were separated in a 1.5% agarose gel, stained with ethidium bromide (EtBr; Sigma-Aldrich, St. Louis, MO, USA), and analyzed with Gel Doc 2000 (Bio-Rad Laboratories, Inc., Hercules, CA, USA).

### 3.3. Quantitative Real-Time PCR (qRT-PCR)

To evaluate the expression of factors involved in stem cell migration toward cancer cells, we performed qRT-PCR specific for chemoattractant ligands and receptors expressed by LNCaP prostate cancer, HDF, and therapeutic stem cells. cDNA synthesized from mRNA of the cancer cells, HDF, and stem cells was mixed with 10 μL 2× SYBR green buffer (TaKaRa Co., Ltd.), 0.4 μL 50× ROX reference dye (TaKaRa co., Ltd.), and 200 pM reverse and forward primers specific for uPA/uPAR, SCF/c-Kit, SDF-1/CXCR4, VEGF/VEGFR2, and MCP-1/CCR2. Primer sequences are presented in Table 2. PCR was performed using an Mx3000P thermocycler (Agilent Technologies Inc., Santa Clare, CA, USA) for 40 cycles of denaturation at 95 °C for 15 s, annealing at 58 °C at 15 s, and extension at 72 °C for 15 s. Quantitative results were analyzed as shown. In addition, to measure quantitatively the expression levels of CD gene in both HB1.F3.CD, and HB1.F3.CD.IFN-β cells, cDNAs synthesized from these cells were processed for qRT-PCR according to the method above.

### 3.4. *In vitro* Migration Assay

To measure the stem cell migratory capabilities, conditioned medium (CM) from confluent LNCaP or human fibroblast (control) cells was collected, transferred to 24-well plates (0.8 mL), and incubated at 37 °C for 24 h. Before seeding the stem cells, bottom portions of transwells (8 μm; BD Biosciences, Franklin Lakes, NJ, USA) were coated with fibronectin (250 μg/mL; Sigma-Aldrich) and air-dried. The transwells were inserted into wells containing CM, and each stem cells (1 × 10^5^ cells/well), HB1.F3.CD, HB1.F3.CD.IFN-β, and HB1.F3.CE cells, was seeded in the upper portion of the transwells and cultured at 37 °C for 1 day. To visualize stem cell migration, stem cells in the upper portion of the transwell that had not migrated were scrapping off and fixed in pre-cooled methanol for 20 minutes. The transwells were then stained with a 0.2% crystal violet (Sigma-Aldrich) in 2% methanol for 10 minutes at 37 °C. After washing three times with PBS, the migrated cells were observed with an XI71 inverted microscope (Olympus, Tokyo, Japan).

### 3.5. Cell Proliferation Assay

To test prodrug cytotoxicity, cancer cells were seeded into 96-well plates (5,000 cells/well). Prodrugs of various concentrations, 5-FC (0, 0.01, 0.1, 0.2, 0.3, 0.5, 1.0, and 10.0 mmol/L; Sigma-Aldrich) or CPT-11 (0, 0.1, 0.2, 0.3, 0.4, 0.5, 1.0, and 10.0 mmol/L; Sigma-Aldrich), were then added to the each well and the cells were incubated at 37 °C for 4 days. To measure cell viability, 10 μL MTT solution (10 mg/mL; Sigma-Aldrich) was then added to each well and the plate was incubated for 4 h and formazan crystal was dissolved in dimethyl sulfoxide (99.0% DMSO; Junsei Chemical Co., Ltd., Tokyo, Japan). Absorbance of each well was measured using an ELISA plate reader. To evaluate the antitumor effects of the stem cells, prostate cancer cells along with HB1.F3.CE, HB1.F3.CD, or HB1.F3.CD.IFN-β cells were seeded onto 96-well plates at a density of 5,000 cells/well. The ratio of stem cells to LNCaP cancer cells was 1:2. After 1 day of incubation, prodrugs (CPT-11 or 5-FC) were added to the cultured plates at various concentrations and the cells were incubated for 4 days. Cell viability was analyzed with an MTT assay after 4 days of treatment with the prodrugs. Each experiment was performed in duplicate (*n* = 12).

### 3.6. Statistic Analysis

All data are presented as the mean ± standard deviation (S.D.). All statistical analyses were performed using GraphPad Prism v5.0 (Graphpad Software, Inc., San Diego, CA, USA) with a one-way ANOVA and Tukey’s test. *p-*values < 0.05 were considered statistically significant.

## 4. Discussion and Conclusions

Prostate cancer is one of the most heterogeneous cancers and the most frequently diagnosed cancer in men [29]. In the present study, we used genetically modified stem cells expressing therapeutic genes such as CD, CE, and IFN-β for treating prostate cancer. HB1.F3.CD and HB1.F3.CD.IFN-β cells express the genes for CD and/or IFN-β while HB1.F3.CE cells bear the CE gene. We confirmed the ability of HB1.F3.CD, HB1.F3.CD.IFN-β, or HB1.F3.CE cells to migrate toward LNCaP prostate cancer cells. The tumor-tropic effects of stem cells are generally attributed to growth factors and cytokines [30,31]. Real time-PCR analysis demonstrated that the LNCaP prostate cancer cells expressed several cytokines including VEGF, MCP-1, and SCF. Although all of these ligands were secreted by non-tumorigenic HDF cells, the secreted amount was low in these cells compared to LNCaP prostate cancer cells except MCP-1, and its receptor, CCR2, was expressed at lower levels in the stem cells. On the other hand, LNCaP cells highly secreted VEGF and stem cells also significantly expressed its receptor, VEGFR2. Therefore, the interaction between VEGF and VEGFR2 was considered as a major cause for the tumor-tropic effect of stem cells toward LNCaP prostate cancer cells. Therefore, we believe that interactions between the chemoattractant factors and their receptors may promote stem cell migration toward the LNCaP prostate cancer cells. The chemoattractant factors and the corresponding receptors may thus play an important role in the tumor-tropic migration of the stem cells.

After selectively migrating toward cancer cells, stem cells bearing therapeutic genes can exert their cytotoxic anti-tumor effects at the targeted sites. CD or CE, factors expressed by two types of suicide genes, can convert non-toxic prodrugs (5-FC or CPT-11, respectively) into toxic compounds, 5-FU or SN-38, respectively. In our study, prostate cancer cell viability was significantly inhibited by HB1.F3.CD and HB1.F3.CD.IFN-β cells in the presence of 5-FC. In particular, HB1.F3.CD.IFN-β cells had a powerful anti-tumor effect, demonstrating the “double punch system” involving the simultaneous expression of CD and IFN-β. In a previous study, the combination of IFN-β expression and localized delivery of 5-FU resulted in tumor regression, apoptosis, and improved survival in a hepatocarcinoma animal model [32]. The mechanism underlying the synergistic effect of the “double punch system” involving the CD and IFN-β genes can explained by the fact that 5-FU converted by CD may increase the anti-tumor effect of IFN-β [33].

Next, we measured the anti-tumor effects of HB1.F3.CE cells and the prodrug CPT-11 (Figure 4). In the presence of CPT-11 alone, growth of the LNCaP cells was gradually decreased with increasing concentrations of the drug (0.5~10 mmol/L). This finding indicated that endogenous human CE capable of converting CPT-11 into SN-38 is expressed to a certain degree by these prostate cancer cells. However, treatment with the combination of HB1.F3.CE cells and CPT-11 sharply decreased the number of LNCaP cells with a lower concentration of CPT-11 (0.1 mmol/L). In addition, CPT-11 decreased LNCaP prostate cancer cells by approximately 40% at the concentration of 10 mmol/L, at which HB1.F3.CE cells almost died. This result can be attributed to the effect of CE gene intentionally introduced into the stem cells. On the other hand, in co-culture plates of HB1.F3.CE and LNCaP cells, the cell viability was decreased by roughly 90% at 10 mmol/L of CPT-11. In this study, stem cells and LNCaP prostate cancer cells were co-cultured at the ratio of 2:1 (stem cells 66% : cancer cells 33%) in 96-well plates. Therefore, the viable cells remaining in this co-culture system (about 10% of the initial cell concentration) can be said to be almost the cancer cells. For the cancer cells, the cell viability was decreased from 33% to 10%. In other words, the LNCaP cells died at a rate of 70% in the co-culture system, which means more cell death than in a single culture (40%). Therefore, this co-culture system induced more cancer cell death than a single culture. Our results demonstrated that HB1.F3.CE cells increased the conversion rate of CPT-11 into SN-38, thereby inhibiting cancer cell growth more effectively. Therefore, the application of stem cell-based CE gene therapy has the advantage of reducing the effective prodrug dosage.

In summary, we observed that the stem cells used in our study had an efficient tumor-tropic effect and migrated toward prostate cancer cells at a greater rate compared to non-tumorigenic primary cells. Stem cells expressing the therapeutic CE, CD, and/or IFN-β genes significantly inhibited cancer cells growth in the presence of prodrugs. These results indicate that therapeutic stem cells expressing the CE or CD suicide genes along with IFN-β can provide a new strategy for treating prostate cancer. Our findings need to be further validated by *in vivo* studies using animal models to demonstrate the distinct advantages of stem cell-based therapeutic gene therapies over more conventional chemotherapies.

## Figures and Tables

**Table 1:** Sequences for the semi-quantitative RT-PCR primers and predicted product size.

Gene		Sequence	Predicted product size (bp)
Rabbit CE	reverse	CTCCAGCATCTCTGTGGTGA	237
	forward	TGCTGGGCTATCCACTCTCT	
Human CD	reverse	GCGCGAGTCACCGCCAGCCACCACGGC	559
	forward	GTTTGTAATCGATGGCTTCTGGCTGC	
Human IFN-β	reverse	AAAGAAGCAGCAATTTTCAG	296
	forward	TTTCTCCAGTTTTTCTTCCA	
Human GAPDH	reverse	ATGTTCGTCATGGGTGTGAACCA	351
	forward	TGGCAGGTTTTTCTAGACGGCAG	

**Table 2:** Sequences of the qRT-PCR primers.

Gene		Sequence
**uPA**	reverse	GGCAGGCAGATGGTCTGTAT
	forward	TTGCTCACCACAACGACATT
**uPAR**	reverse	TCCCCTTGCAGCTGTAACACT
	forward	GCCCAATCCTGGAGCTTGA
**SCF**	reverse	GCCTTCAGAAATATTTGAAAACTTG
	forward	GGCAAATCTTCCAAAAGACTACA
**c-Kit**	reverse	TCACAGATGGTTGAGAAGAGCCT
	forward	CGCCTGGGATTTTCTCTGC
**SDF-1**	reverse	TCCCATCCCACAGAGAGAAG
	forward	GTGTCACTGGCGACACGTAG
**CXCR4**	reverse	GAGGGCCTTGCGCTTCTGGTG
	forward	ATCCCTGCCCTCCTGCTGACTATTC
**VEGF**	reverse	TCTTTCTTTGGTCTGCATTCACAT
	forward	CCAGCACATAGGAGAGATGAGCTT
**VEGFR2**	reverse	AGCATGGAAGAGGATTCTGGACT
	forward	CGGCTCTTTCGCTTACTGTTCT
**MCP-1**	reverse	TCTTCGGAGTTTGGGTTTGC
	forward	CAAGCAGAAGTGGGTTCAGGA
**CCR-2**	reverse	ACATTTACAAGTTGCAGTTTTCAGC
	forward	CTACCTTCCAGTTCCTCATTTTT
